# 3-Chloro-6-(1*H*-pyrazol-1-yl)pyridazine

**DOI:** 10.1107/S1600536810027121

**Published:** 2010-07-14

**Authors:** Abdul Qayyum Ather, M. Nawaz Tahir, Misbahul Ain Khan, Muhammad Makshoof Athar, Eliana AparecidaSilicz Bueno

**Affiliations:** a Department of Chemistry, Islamia University, Bahawalpur, Pakistan; b Applied Chemistry Research Center, PCSIR Laboratories Complex, Lahore 54600, Pakistan; c Department of Physics, University of Sargodha, Sargodha, Pakistan; d Institute of Chemistry, University of the Punjab, Lahore, Pakistan; e Instituto de Quimica, Universidade Estadual de Londrina, Londrina, Pr., Brazil

## Abstract

The title compound, C_7_H_5_ClN_4_, is almost planar (r.m.s. deviation = 0.022 Å). The dihedral angle between the aromatic rings is 2.82 (5)°. The packing results in polymeric chains extending along the *a* axis. In the crystal, mol­ecules are connected to each other through inter­molecular C—H⋯N hydrogen bonds, resulting in *R*_2_^2^(10) ring motifs.

## Related literature

For related structures, see: Ather *et al.* (2009 ▶, 2010*a* ▶,*b* ▶). For graph-set notation, see: Bernstein *et al.* (1995 ▶).
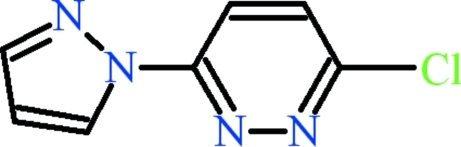


## Experimental

### 

#### Crystal data

C_7_H_5_ClN_4_
*M* *_r_* = 180.60
Triclinic, 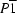

*a* = 5.684 (3) Å
*b* = 6.526 (3) Å
*c* = 11.130 (6) Å
α = 83.00 (3)°
β = 77.64 (2)°
γ = 88.04 (3)°
*V* = 400.2 (4) Å^3^
*Z* = 2
Mo *K*α radiation
μ = 0.42 mm^−1^
*T* = 296 K
0.30 × 0.14 × 0.14 mm

#### Data collection

Bruker Kappa APEXII CCD diffractometer
Absorption correction: multi-scan (*SADABS*; Bruker, 2005 ▶) *T*_min_ = 0.982, *T*_max_ = 0.988
5550 measured reflections
1422 independent reflections
774 reflections with *I* > 2σ(*I*)
*R*_int_ = 0.071

#### Refinement

*R*[*F* ^2^ > 2σ(*F* ^2^)] = 0.062
*wR*(*F* ^2^) = 0.154
*S* = 1.08
1422 reflections
109 parameters
H-atom parameters constrained
Δρ_max_ = 0.22 e Å^−3^
Δρ_min_ = −0.22 e Å^−3^

### 

Data collection: *APEX2* (Bruker, 2007 ▶); cell refinement: *SAINT* (Bruker, 2007 ▶); data reduction: *SAINT*; program(s) used to solve structure: *SHELXS97* (Sheldrick, 2008 ▶); program(s) used to refine structure: *SHELXL97* (Sheldrick, 2008 ▶); molecular graphics: *ORTEP-3 for Windows* (Farrugia, 1997 ▶) and *PLATON* (Spek, 2009 ▶); software used to prepare material for publication: *WinGX* (Farrugia, 1999 ▶) and *PLATON*.

## Supplementary Material

Crystal structure: contains datablocks global, I. DOI: 10.1107/S1600536810027121/vm2034sup1.cif

Structure factors: contains datablocks I. DOI: 10.1107/S1600536810027121/vm2034Isup2.hkl

Additional supplementary materials:  crystallographic information; 3D view; checkCIF report

## Figures and Tables

**Table 1:** Hydrogen-bond geometry (Å, °)

*D*—H⋯*A*	*D*—H	H⋯*A*	*D*⋯*A*	*D*—H⋯*A*
C3—H3⋯N2^i^	0.93	2.46	3.321 (6)	154
C5—H5⋯N4^ii^	0.93	2.55	3.468 (7)	169

Symmetry codes: (i) 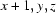; (ii) 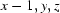.

**Fig. 1.**
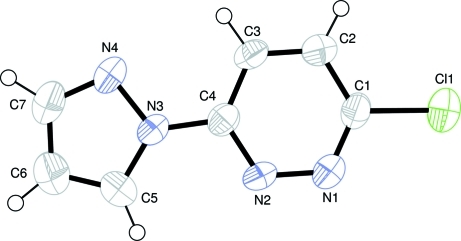
View of the title compound with the atom numbering scheme. The thermal ellipsoids are drawn at the 50% probability level. H-atoms are shown as small spheres of arbitrary radii.

**Fig. 2.**
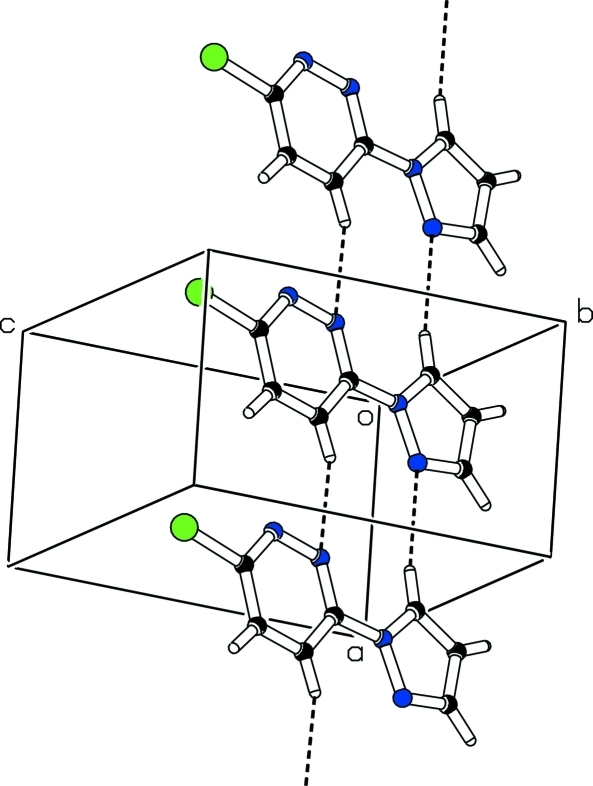
Packing diagram of the title compound (PLATON: Spek, 2009) showing that the molecules form polymeric chains extending along the a axis.

## supplementary crystallographic information

### Comment

The title compound (I, Fig. 1) has been prepared as nucleous to synthesize a series of pyrazolylpyridazine derivatives. In this context, we have already reported some compounds (Ather *et al.*, 2009, 2010*a*, 2010*b*).

The title compound is essentially planar. The r. m. s. deviation of all heavy atoms from the mean square plane is 0.0223 Å. The angle between the heterocyclic aromatic rings is 2.82 (5)°. Each molecule is connected to the adjacent molecules through C—H···N intermolecular H-bonds (Table 1, Fig. 2) and *R*_2_^2^(10) ring motifs (Bernstein *et al.*, 1995) are formed. In this way the packing forms one dimensional polymeric chains extending along the crystallographic *a* axis.

### Experimental

3-Chloro-6-hydrazinylpyridazine (0.5 g, 3.46 mmol) was dissolved in ethanol (15 ml) and malon dialdehyde bis-(diethylacetal) (0.327 g, 3.46 mmol) was added dropwise under continuous stirring. Few drops of acetic acid were also added in the reaction mixture as catalyst and the solution was refluxed for 2 h. The reaction was monitored by TLC. After completion, the reaction mixture was precipitated by adding water. The filtered precipitate was dried and colorless needles of (I) appeared on the walls of the beaker due to evaporation.

### Refinement

Although all H-atoms appear in the difference Fourier map they were positioned geometrically (C–H = 0.93 Å) and refined as riding atoms with *U*_iso_(H) = 1.2*U*_eq_(C).

### Crystal data

**Table:**

C_7_H_5_ClN_4_	*Z* = 2
*M**_r_* = 180.60	*F*(000) = 184
Triclinic, *P*1	*D*_x_ = 1.499 Mg m^−^^3^
Hall symbol: -P 1	Mo *K*α radiation, λ = 0.71073 Å
*a* = 5.684 (3) Å	Cell parameters from 774 reflections
*b* = 6.526 (3) Å	θ = 3.2–25.1°
*c* = 11.130 (6) Å	µ = 0.42 mm^−^^1^
α = 83.00 (3)°	*T* = 296 K
β = 77.64 (2)°	Needle, colourless
γ = 88.04 (3)°	0.30 × 0.14 × 0.14 mm
*V* = 400.2 (4) Å^3^	

### Data collection

**Table:**

Bruker Kappa APEXII CCD diffractometer	1422 independent reflections
Radiation source: fine-focus sealed tube	774 reflections with *I* > 2σ(*I*)
graphite	*R*_int_ = 0.071
Detector resolution: 8.20 pixels mm^-1^	θ_max_ = 25.1°, θ_min_ = 3.2°
ω scans	*h* = −6→6
Absorption correction: multi-scan (*SADABS*; Bruker, 2005)	*k* = −7→7
*T*_min_ = 0.982, *T*_max_ = 0.988	*l* = −13→13
5550 measured reflections	

### Refinement

**Table:**

Refinement on *F*^2^	Primary atom site location: structure-invariant direct methods
Least-squares matrix: full	Secondary atom site location: difference Fourier map
*R*[*F*^2^ > 2σ(*F*^2^)] = 0.062	Hydrogen site location: inferred from neighbouring sites
*wR*(*F*^2^) = 0.154	H-atom parameters constrained
*S* = 1.08	*w* = 1/[σ^2^(*F*_o_^2^) + (0.0381*P*)^2^ + 0.276*P*] where *P* = (*F*_o_^2^ + 2*F*_c_^2^)/3
1422 reflections	(Δ/σ)_max_ < 0.001
109 parameters	Δρ_max_ = 0.22 e Å^−^^3^
0 restraints	Δρ_min_ = −0.22 e Å^−^^3^

### Special details

**Table:**

Geometry. Bond distances, angles *etc*. have been calculated using the rounded fractional coordinates. All su's are estimated from the variances of the (full) variance-covariance matrix. The cell e.s.d.'s are taken into account in the estimation of distances, angles and torsion angles
Refinement. Refinement of *F*^2^ against ALL reflections. The weighted *R*-factor *wR* and goodness of fit *S* are based on *F*^2^, conventional *R*-factors *R* are based on *F*, with *F* set to zero for negative *F*^2^. The threshold expression of *F*^2^ > σ(*F*^2^) is used only for calculating *R*-factors(gt) *etc*. and is not relevant to the choice of reflections for refinement. *R*-factors based on *F*^2^ are statistically about twice as large as those based on *F*, and *R*- factors based on ALL data will be even larger.

### Fractional atomic coordinates and isotropic or equivalent isotropic displacement parameters (Å^2^)

**Table:**

	*x*	*y*	*z*	*U*_iso_*/*U*_eq_	
Cl1	0.0311 (2)	0.6902 (2)	0.86426 (11)	0.0772 (6)	
N1	−0.0066 (6)	0.7294 (6)	0.6368 (3)	0.0586 (14)	
N2	0.0752 (6)	0.7486 (6)	0.5126 (3)	0.0525 (14)	
N3	0.3753 (6)	0.7757 (5)	0.3384 (3)	0.0449 (14)	
N4	0.6127 (6)	0.7910 (6)	0.2806 (3)	0.0576 (16)	
C1	0.1521 (8)	0.7182 (7)	0.7066 (4)	0.0503 (17)	
C2	0.4010 (7)	0.7237 (7)	0.6628 (4)	0.0506 (17)	
C3	0.4819 (7)	0.7451 (6)	0.5399 (4)	0.0450 (16)	
C4	0.3094 (7)	0.7556 (6)	0.4674 (4)	0.0417 (16)	
C5	0.2316 (9)	0.7798 (8)	0.2556 (4)	0.0676 (19)	
C6	0.3770 (10)	0.7966 (9)	0.1421 (5)	0.078 (2)	
C7	0.6095 (9)	0.8042 (8)	0.1617 (5)	0.0701 (19)	
H2	0.50643	0.71294	0.71672	0.0608*	
H3	0.64564	0.75254	0.50460	0.0538*	
H5	0.06440	0.77251	0.27366	0.0814*	
H6	0.33112	0.80200	0.06646	0.0928*	
H7	0.74672	0.81708	0.09857	0.0841*	

### Atomic displacement parameters (Å^2^)

**Table:**

	*U*^11^	*U*^22^	*U*^33^	*U*^12^	*U*^13^	*U*^23^
Cl1	0.0676 (9)	0.1105 (13)	0.0459 (8)	−0.0045 (8)	0.0038 (6)	−0.0068 (7)
N1	0.040 (2)	0.084 (3)	0.047 (2)	−0.002 (2)	0.0002 (19)	−0.005 (2)
N2	0.028 (2)	0.076 (3)	0.051 (2)	−0.0027 (17)	−0.0050 (16)	−0.003 (2)
N3	0.0321 (19)	0.055 (3)	0.044 (2)	−0.0020 (17)	−0.0012 (16)	−0.0033 (18)
N4	0.041 (2)	0.076 (3)	0.049 (3)	−0.0033 (19)	0.0048 (17)	−0.005 (2)
C1	0.046 (3)	0.061 (3)	0.040 (3)	−0.004 (2)	0.002 (2)	−0.009 (2)
C2	0.040 (3)	0.060 (3)	0.052 (3)	−0.001 (2)	−0.011 (2)	−0.005 (2)
C3	0.031 (2)	0.049 (3)	0.052 (3)	−0.0045 (19)	−0.004 (2)	−0.001 (2)
C4	0.039 (2)	0.038 (3)	0.046 (3)	0.0016 (19)	−0.003 (2)	−0.008 (2)
C5	0.058 (3)	0.097 (4)	0.051 (3)	−0.002 (3)	−0.021 (3)	−0.004 (3)
C6	0.079 (4)	0.104 (5)	0.050 (3)	−0.009 (3)	−0.015 (3)	−0.006 (3)
C7	0.061 (3)	0.089 (4)	0.051 (3)	−0.008 (3)	0.008 (2)	−0.005 (3)

### Geometric parameters (Å, °)

**Table:**

Cl1—C1	1.732 (5)	C2—C3	1.338 (6)
N1—N2	1.353 (5)	C3—C4	1.392 (6)
N1—C1	1.306 (6)	C5—C6	1.348 (7)
N2—C4	1.319 (5)	C6—C7	1.388 (8)
N3—N4	1.366 (5)	C2—H2	0.9300
N3—C4	1.395 (5)	C3—H3	0.9300
N3—C5	1.354 (6)	C5—H5	0.9300
N4—C7	1.320 (6)	C6—H6	0.9300
C1—C2	1.394 (6)	C7—H7	0.9300
			
Cl1···Cl1^i^	3.632 (3)	C3···N2^vi^	3.321 (6)
Cl1···H7^ii^	2.9500	C3···C3^iv^	3.411 (6)
N1···C2^iii^	3.318 (6)	C3···C3^v^	3.339 (6)
N1···C3^iii^	3.305 (6)	C3···C4^iv^	3.442 (6)
N2···C3^iii^	3.321 (6)	C3···C4^v^	3.491 (6)
N4···C2^iv^	3.342 (6)	C4···C3^iv^	3.442 (6)
N4···C2^v^	3.299 (6)	C4···C3^v^	3.491 (6)
N1···H2^iii^	2.7200	C7···H5^vi^	3.0900
N1···H3^iii^	2.7000	H2···N1^vi^	2.7200
N2···H3^iii^	2.4600	H3···N1^vi^	2.7000
N2···H5	2.6600	H3···N2^vi^	2.4600
N4···H5^vi^	2.5500	H3···N4	2.5200
N4···H3	2.5200	H5···N2	2.6600
C2···N1^vi^	3.318 (6)	H5···N4^iii^	2.5500
C2···N4^iv^	3.342 (6)	H5···C7^iii^	3.0900
C2···N4^v^	3.299 (6)	H7···Cl1^vii^	2.9500
C3···N1^vi^	3.305 (6)		
			
N2—N1—C1	117.9 (4)	N3—C5—C6	106.9 (5)
N1—N2—C4	119.1 (3)	C5—C6—C7	105.7 (5)
N4—N3—C4	120.1 (3)	N4—C7—C6	112.0 (5)
N4—N3—C5	111.4 (3)	C1—C2—H2	121.00
C4—N3—C5	128.5 (4)	C3—C2—H2	121.00
N3—N4—C7	104.0 (4)	C2—C3—H3	122.00
Cl1—C1—N1	114.7 (3)	C4—C3—H3	122.00
Cl1—C1—C2	120.4 (3)	N3—C5—H5	126.00
N1—C1—C2	124.9 (4)	C6—C5—H5	127.00
C1—C2—C3	117.2 (4)	C5—C6—H6	127.00
C2—C3—C4	116.9 (4)	C7—C6—H6	127.00
N2—C4—N3	114.7 (4)	N4—C7—H7	124.00
N2—C4—C3	124.0 (4)	C6—C7—H7	124.00
N3—C4—C3	121.3 (4)		
			
C1—N1—N2—C4	−0.1 (6)	N4—N3—C5—C6	0.4 (6)
N2—N1—C1—Cl1	−179.1 (3)	C4—N3—C5—C6	−178.6 (4)
N2—N1—C1—C2	−0.3 (7)	N3—N4—C7—C6	−0.3 (6)
N1—N2—C4—N3	−180.0 (4)	Cl1—C1—C2—C3	179.8 (3)
N1—N2—C4—C3	−0.2 (6)	N1—C1—C2—C3	1.0 (7)
C4—N3—N4—C7	179.0 (4)	C1—C2—C3—C4	−1.3 (6)
C5—N3—N4—C7	0.0 (5)	C2—C3—C4—N2	1.0 (6)
N4—N3—C4—N2	178.0 (4)	C2—C3—C4—N3	−179.3 (4)
N4—N3—C4—C3	−1.7 (6)	N3—C5—C6—C7	−0.6 (6)
C5—N3—C4—N2	−3.1 (6)	C5—C6—C7—N4	0.6 (7)
C5—N3—C4—C3	177.2 (4)		

Symmetry codes: (i) −*x*, −*y*+1, −*z*+2; (ii) *x*−1, *y*, *z*+1; (iii) *x*−1, *y*, *z*; (iv) −*x*+1, −*y*+1, −*z*+1; (v) −*x*+1, −*y*+2, −*z*+1; (vi) *x*+1, *y*, *z*; (vii) *x*+1, *y*, *z*−1.

### Hydrogen-bond geometry (Å, °)

**Table:**

*D*—H···*A*	*D*—H	H···*A*	*D*···*A*	*D*—H···*A*
C3—H3···N2^vi^	0.9300	2.4600	3.321 (6)	154.00
C5—H5···N4^iii^	0.9300	2.5500	3.468 (7)	169.00

Symmetry codes: (vi) *x*+1, *y*, *z*; (iii) *x*−1, *y*, *z*.
